# The Oldest Tree in the World

**Published:** 1883-08

**Authors:** 


### THE OLDEST TREE IN THE WORLD.

The oldest tree in the world, so far as any one knows, is, says *Knowledge*, the Bo tree, of the sacred city of Amarapoora, in Burmah. It was planted 288 B. C., and is therefore now 2,170 years old. Sir James Emerson Tennent gives reasons for believing that the tree is really of this wonderful age, and refers to historic documents in which it is mentioned at different dates, as 182 A. D., and so on to the present day. "To it," says Sir James, "kings have even dedicated their dominions, in testimony of a belief that it is a branch of the identical fig tree under which Buddha reclined at Urumelaya when he underwent his apotheosis." Its leaves are carried away as streamers by pilgrims, but it is too sacred to touch with a knife, and therefore they are only gathered when they fall. The King Oak in Windsor Forest, England, is 1,000 years old.

